# Seabird diving behaviour reveals the functional significance of shelf-sea fronts as foraging hotspots

**DOI:** 10.1098/rsos.160317

**Published:** 2016-09-21

**Authors:** S. L. Cox, P. I. Miller, C. B. Embling, K. L. Scales, A. W. J. Bicknell, P. J. Hosegood, G. Morgan, S. N. Ingram, S. C. Votier

**Affiliations:** 1 Marine Biology and Ecology Research Centre, Plymouth University, Plymouth PL4 8AA, UK; 2 Marine Physics Research Group, Plymouth University, Plymouth PL4 8AA, UK; 3 Plymouth Marine Laboratory, Prospect Place, Plymouth PL1 3DH, UK; 4 Institute of Marine Sciences, University of California, Santa Cruz, CA 95064, USA; 5 National Oceanic and Atmospheric Administration (NOAA), Southwest Fisheries Science Centre, Environmental Research Division, 99 Pacific Street, Suite 255A, Monterey, CA 93940, USA; 6 Environment and Sustainability Institute, University of Exeter, Penryn TR10 9FE, UK; 7 RSPB, Ramsey Island, St David's, Pembrokeshire SA62 6PY, UK

**Keywords:** seabird, marine predator, oceanography, ocean front, diving behaviour, foraging ecology

## Abstract

Oceanic fronts are key habitats for a diverse range of marine predators, yet how they influence fine-scale foraging behaviour is poorly understood. Here, we investigated the dive behaviour of northern gannets *Morus bassanus* in relation to shelf-sea fronts. We GPS (global positioning system) tracked 53 breeding birds and examined the relationship between 1901 foraging dives (from time-depth recorders) and thermal fronts (identified via Earth Observation composite front mapping) in the Celtic Sea, Northeast Atlantic. We (i) used a habitat-use availability analysis to determine whether gannets preferentially dived at fronts, and (ii) compared dive characteristics in relation to fronts to investigate the functional significance of these oceanographic features. We found that relationships between gannet dive probabilities and fronts varied by frontal metric and sex. While both sexes were more likely to dive in the presence of seasonally persistent fronts, links to more ephemeral features were less clear. Here, males were positively correlated with distance to front and cross-front gradient strength, with the reverse for females. Both sexes performed two dive strategies: shallow V-shaped plunge dives with little or no active swim phase (92% of dives) and deeper U-shaped dives with an active pursuit phase of at least 3 s (8% of dives). When foraging around fronts, gannets were half as likely to engage in U-shaped dives compared with V-shaped dives, independent of sex. Moreover, V-shaped dive durations were significantly shortened around fronts. These behavioural responses support the assertion that fronts are important foraging habitats for marine predators, and suggest a possible mechanistic link between the two in terms of dive behaviour. This research also emphasizes the importance of cross-disciplinary research when attempting to understand marine ecosystems.

## 1. Introduction

Large marine predators, such as marine mammals, seabirds, turtles and sharks, forage over long distances in dynamic environments where prey are patchily distributed [1,2]. Many of these predators display targeted and individually consistent movement patterns [3–6], that are frequently linked to physical oceanographic features, including fronts [7–9], eddies [7,10], tidal flow fields [11] and regions of stratification [12], where low- to mid-trophic-level prey accessibility and availability is enhanced [13,14].

Ocean fronts are important habitats for an array of taxonomically diverse marine predators [7–9]. Fronts are physical structures, occurring between adjacent water masses of differing properties that produce strong gradients in density, temperature and/or salinity [15]. Physical attributes of fronts promote enhanced primary productivity [16,17] and biomass accumulation/redistribution [18], often in a predictable manner. This may have bottom-up effects that propagate across multiple trophic levels, resulting in the formation of dense, and sometimes shallow, aggregations of prey [19–21] that may be important for upper trophic-level consumers [22]. However, while such mechanisms are often posited to explain why fronts make attractive foraging habitats, to date, such links have yet to be fully explored. This shortfall is mostly owing to the logistical challenges of simultaneously measuring oceanography, lower- to mid-trophic-level prey and large marine predators at appropriate spatio-temporal scales.

Interactions between predators and oceanography have been predominantly revealed through the analysis of two-dimensional horizontal animal movement data obtained either directly from shipboard observations [23] or remotely through animal-borne telemetry [24]. However, for diving predators, much can be learnt through understanding vertical movements, particularly in species that employ different dive strategies. Specifically, changes in three-dimensional predator behaviours may be coupled to the fine-scale horizontal and vertical distributions of their prey [25–27]. As such, understanding how large marine predators respond to fronts in the vertical dimension may help us resolve the functional mechanisms that link the two.

Advances in biologging technologies mean we are now able to observe fine-scale animal movements in three dimensions across entire foraging trips [28]. These data can be supplemented with near real-time remotely sensed information on biological and physical oceanography. However, the relevance of traditionally used measurements such as sea surface temperature (SST) and surface chlorophyll *a* concentration has, at times, proved questionable, and these oceanographic descriptors are not always good at predicting the distributions of marine predators [29,30]. Composite front mapping [31–33] is a relatively new technique that attempts to address this by objectively identifying discrete oceanographic frontal features that are, *a priori*, thought to represent prosperous foraging habitats. In addition, features are quantified through the output of several front metric products, that can be derived over a number of spatio-temporal scales [32,34] allowing the dynamic nature of a front to be characterized [8,35] while also overcoming problems of cloud obfuscating signal. This may be particularly useful in highly dynamic environments where passing ephemeral activity can weaken links to marine predators [35].

In this study, we used the northern gannet, *Morus bassanus,* to investigate the influence of shelf-sea fronts on the distributions and characteristics of dives. We used bird-borne global positioning system (GPS) loggers and time-depth recorders (TDRs), deployed on centrally placed breeders from a large colony in the Celtic Sea (Grassholm, Wales, UK), to link dive events with multiple products derived from weekly and seasonal composite front maps. Specifically we asked: (i) do gannets preferentially dive around fronts, (ii) do gannets change dive strategy (i.e. dive shape) around fronts and (iii) do the depths and durations of dives decrease around fronts?

Gannets are large, medium-ranging piscivorous marine predators [5,36] that exhibit a nested search strategy, and dive infrequently, presumably only when prey have been located [5,37]. Foraging strategies range from short shallow plunge dives, to longer and deeper wing-propelled active pursuit dives that can reach up to 25 m in depth [37,38]. Gannets in the Celtic Sea feed on a variety of forage and pelagic fish such as mackerel *Scomber scombrus*, garfish *Belone belone*, herring *Clupea harengus* and sprat *Sprattus sprattus*, and a range of demersal fishes, scavenged from fishing boats [39–41]. Previous work has shown that both in the Celtic Sea and the Benguela upwelling region off western South Africa, gannets increase foraging effort (estimated via two-dimensional movement data) within regions where frontal activity is increased [35,42]. By examining their diving behaviour, we attempt to establish why.

## 2. Material and methods

### 2.1. Device deployment

**Figure 1.**
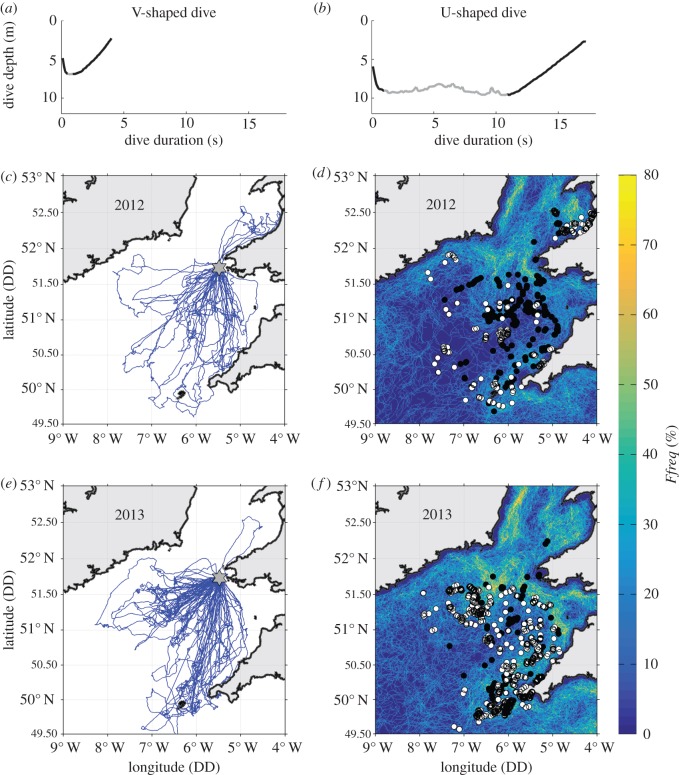
Rows show, from top to bottom: typical V- (*a*) and U-shaped (*b*), dive profiles with defined active swim phase highlighted in grey, 2012 GPS tracks (*c*) and associated dives overlaid on *Ffreq* (*d*), and 2013 GPS tracks (*e*) and associated dives overlaid on *Ffreq* (*f*). Black and white dive markers (in subplots *d*,*f*) represent male and female dives, respectively. The location of Grassholm corresponds to the grey star on the GPS track plots (*c*,*e*).

Fieldwork was conducted on Grassholm, Wales, UK (51°43′ N, 5°28′ W; figure 1), during the breeding season in July 2012 and 2013. Chick-rearing gannets were caught on the nest during changeover (to ensure chicks were not left unattended, and so deployments began immediately with a foraging trip), using a brass crook attached to the end of an approximately 5 m carbon fibre pole. Birds were selected opportunistically, away from the edge of the colony (under licence from Natural Resources Wales). Upon capture, birds were weighed (to the nearest 50 g) and 1–2 ml of blood taken via the tarsal vein (under licence from the UK Home Office), a small aliquot of which was later used for molecular sexing (commercially outsourced to AvianBiotech.com). Birds were then equipped, using Tesa® tape, with (i) a 30 g GPS logger (i-gotU GT-120, Mobile Active Technology Inc.) attached to either the dorsal surface of the central pair of tail feathers (2012) or the central back feathers (2013) and (ii) a 5.7 g or 10.5 g TDR (CEFAS G5 or LOTEK LAT 1810, respectively) attached to the ventral surface of the central pair of tail feathers (under licence from the British Trust for Ornithology). Total handling time was around 12 min. The maximum combined weight of deployed loggers (40.5 g) was 1.37% the average bird body weight (2948.8 g ± 33.0 g). Deployment durations ranged from 1 to 7 days.

### 2.2. Particulars of device data acquisition

The GPS recorder logged location at 1 min intervals with an accuracy of ±4.4 m [43]. The G5 TDR logged pressure and temperature every 0.1 s (10 Hz) during dives, defined as wet periods (detected via a wet/dry sensor) below a depth of 1.5 m. Pressure resolution was 4 cm of water column with an accuracy of ±1 m. The LOTEK LAT 1810 TDR logged temperature and pressure continuously at 1 s intervals (1 Hz). Pressure resolution was 2.5 cm of water column with an accuracy of ±1 m.

### 2.3. Animal behaviour metrics

#### 2.3.1. Global positioning system tracks

GPS fixes at night (between the end of civil dusk to the beginning of civil dawn) were stripped from the dataset to eliminate periods when birds rest on the water [44,45]. In addition, all activity within 2 km of the breeding colony was removed to account for bathing and rafting [46]. Tracks were then split into individual foraging trips. In some instances, the device deployment period exceeded the battery life of the GPS logger. Resultant incomplete foraging trips were included in analyses, unless otherwise stated.

#### 2.3.2. Dive events

A bespoke algorithm, written in Matlab, was used to identify dive events by birds equipped with a LOTEK TDR. To be consistent with the technicalities of the CEFAS logger, dives were defined as periods where the registered depth was at least 1.5 m. This also accounted for shallow subsurface activity associated with non-foraging behaviours such as sitting on the water and bathing [38,47].

To allocate a location for each dive, GPS tracks were first interpolated to a resolution of 1 s (in time), using a cubic spline interpolation. The timestamp at the beginning of a dive event was then used to assign a corresponding location from the high-resolution interpolation. Allocations mismatched by more than a second (owing to the GPS logger battery life being surpassed or because a dive occurred within the colony exclusion zone) were excluded from analyses. A small number of shallow dives (five in total) occurred approximately 40 min after the end of civil dusk. These were considered atypical and excluded from analyses [44,45].

#### 2.3.3. Dive characteristics

Gannets predominantly employ two dive strategies (figure 1*a*,*b*). Short and typically shallow plunge dives (termed V-shaped; figure 1*a*) involve little or no active swim phase [38,48], and may be used to catch fast, responsive prey in the upper section of the water column [49–51]. Indeed, this strategy is often associated with the predation of near-surface pelagic fish such as herring and mackerel [49,50]. In contrast, longer deeper pursuit dives (U-shaped; figure 1*b*) involve a substantial active swim phase that likely incurs higher energetic costs [37,52]. As such, this foraging strategy may only be used following a failed plunge attempt [37,48], or when prey are distributed at deeper depths (e.g. shoals of capelin *Mallotus villosus* or sand eels *Ammodytes* sp. at depth and/or near the seabed; [49,51]). In some instances, these dives may also be used to catch multiple prey items [51], although this is likely to be dependent upon the responsiveness and swimming speeds of prey [49,50]. To be able to examine if/how fronts influence dive strategy (which may reflect changes in prey accessibility and catchability), each dive was classified as either U- or V-shaped (figure 1*a*,*b*), as determined by the length of the active swim phase (U-shaped dives were those with an active swim phase of at least 3 or at least 4 s dependent upon the logger sampling rate; 1 s and 10 Hz, respectively; [38]). The start and end of the active swim phase was defined, using gradients in the vertical change in depth (electronic supplementary material, S1 and figure S1; [37,53]). In addition, for each dive, the maximum dive depth (metres) and total dive duration (seconds) was calculated.

### 2.4. Front metrics

Fronts vary in their strength, persistence and predictability [8]. To be able to determine the relative importance of these characteristics on foraging behaviour, thermal front activity across the Celtic Sea was summarized into three metrics that reflected this variability: (i) cross-front gradient strength *Gdens*, (ii) distance to closest front *Fdist*, and (iii) seasonal front frequency *Ffreq*. These were produced over two temporal scales: (i) 7 day composites [31] and (ii) seasonal composites [32], details of which are provided below (see §2.4.1 Seven day composites and §2.4.2 Seasonal composites). As the occurrences of thermal and chlorophyll fronts are typically linked across shelf-seas (e.g. at tidal-mixing fronts; [34,54]), we did not include extra analyses, using chlorophyll *a* derived front metrics in this study.

#### 2.4.1. Seven day composites

**Figure 2.**
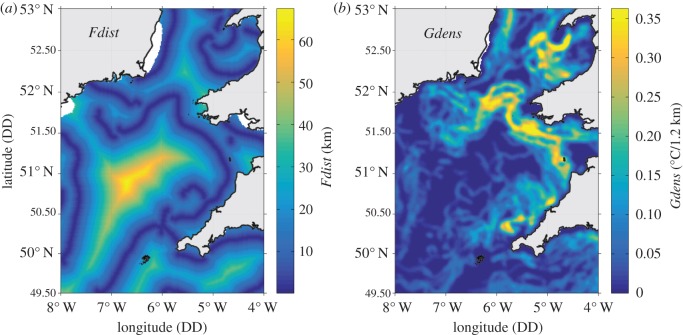
Front metrics derived for the Celtic Sea over 7 day composites are shown for the 21 July 2013. From left to right, (*a*) *Fdist* (distance to closet front) and (*b*) *Gdens* (cross-front gradient strength).

*Gdens* and *Fdist* were based on 7 day composite front maps centred to the date of a GPS/TDR fix (figure 2). First, raw (level 0) advanced very high-resolution radiometer (AVHRR) infrared data were converted to an index of SST (level 2). SST data were then mapped across the Celtic Sea with a spatial resolution of approximately 1.2 km^2^/pixel. Thermal fronts were detected over frames of 32 × 32 pixels, using single image edge detection (SIED; [55]) with a temperature difference threshold of 0.4°C across the front [31] comparable to [35]. The SIED front map generated from a single satellite image is unsuitable for the description of fronts owing to cloud cover in the study region. Therefore, all frontal segments obtained during the 7 day window were combined to obtain a more synoptic frontal picture [31]. If cloud persisted for the entire 7 days in certain regions, then these were marked as missing in the front metrics, and any corresponding bird tracks were excluded from analysis.

*Gdens* was then taken as the spatially smoothed average temperature gradient across all frontal pixels detected over a 7 day composite to give a continuous distribution of frontal intensity. A Gaussian filter with a width of five pixels was used for the spatial smoothing [34,35]. This metric indicates the intensity of contemporaneous frontal structures that may be either transient or persistent in occurrence.

*Fdist* was taken as the distance from any point to the closest simplified front. Simplified fronts were defined, using a clustering algorithm to identify continuous contours through the strongest frontal pixels on the spatially smoothed *Gdens* front map [35]. This metric quantifies the distance from each dive/pseudo-absence location (see §2.5 Statistical analysis) to the continuous and discrete surface signature of more defined frontal systems, and generally excludes the smaller ephemeral features that are often picked up by the *Gdens* metric.

#### 2.4.2. Seasonal composites

*Ffreq* was based on all front maps generated between June and August inclusive for each year (2012 and 2013) separately (figure 1). Maps generated before spatial smoothing were used, and *Ffreq* was taken as the percentage of total detections in which a frontal temperature gradient greater than or equal to 0.04°C was observed (see *Average front gradient* in [31]). This threshold reduced noise in front detections associated with minor discrepancies in temperature observations [32]. This metric indicates areas where fronts frequently manifest across a season and so are generally persistent and highly predictable in occurrence.

### 2.5. Statistical analysis

Three modelling approaches were used to investigate the influence of our three front metrics (*Gdens*, *Fdist* and *Ffreq*), on gannet dive behaviour (table 1). First, habitat use versus availability was modelled against frontal activity. This was achieved using generalized linear mixed effects models (GLMMs) from the MASS package in R [56] with a binomial error structure and complementary log–log (cloglog) link function [57,58]. For each dive event, the locations of five pseudo-absences were randomly selected from within the bounds of the 95% utilization distribution of the population sample (see electronic supplementary material, S2 and figure S2; [59]). Second, dive shape was modelled against frontal activity. This approach also used GLMMs from the MASS package in R [56] with a binomial error structure and complementary log–log (cloglog) link function. Finally, the influence of frontal activity on dive depth and duration was modelled separately for U- and V-shaped dives using linear mixed effects models (LMMs) from the R package nlme [60].

**Table 1.** Overview of the three modelling approaches applied to determine the influence of frontal activity on (*a*) habitat usage and the probability of a dive event, (*b*) dive shape (U versus V) and (*c*) depth and duration of U- and V-shaped dives. Terms between the curly brackets are interchangeable to represent where models were fitted separately to avoid issues associated with multicollinearity. The initial fixed component with all potential explanatory variables (before model reduction/selection) is shown. The random component comprises a random intercept of *BirdID* and either (i) a nested spatial correlation structure *CorStructSp* (*a*) or (ii) a nested continuous temporal correlation structure *CorStructTp* (*b,c*).

(*a*) Generalized linear mixed effects model (GLMM) with binomial error structure and complementary log–log (cloglog) link function
$\text{Dive event}\;(0/1)\;∼\overset{\mathrm{Fixed}\;\mathrm{component}}{\overbrace{\left\{\begin{array}{c} \text{Gdens}\\ \text{Fdist}\\ \text{Ffreq} \end{array}\right\}\times \text{Sex}}}+\overset{\mathrm{Random}\;\mathrm{component}}{\overbrace{\left(1|\text{BirdID})+(\text{CorStructSp}|\text{BirdID}\right)}}$
(*b*) GLMM with binomial error structure and complementary log–log (cloglog) link function
$\text{Dive shape}\;(0/1)\;∼\overset{\mathrm{Fixed}\;\mathrm{component}}{\overbrace{\left\{\begin{array}{c} \text{Gdens}\\ \text{Fdist}\\ \text{Ffreq} \end{array}\right\}\times \text{Sex}+\text{DecTime}+{\text{DecTime}}^{2}}}+\overset{\mathrm{Random}\;\mathrm{component}}{\overbrace{\left(1|\text{BirdID})+(\text{CorStructTp}|\text{BirdID}\right)}}$
(*c*) linear mixed effects model (LMM)
$\left\{\begin{array}{c} U\text{dive depth}(m)\\ V\text{dive depth}(m)\\ U\text{dive duration}(s)\\ V\text{dive duration}(s) \end{array}\right\}∼\overset{\mathrm{Fixed}\;\mathrm{component}}{\overbrace{\left\{\begin{array}{c} \text{Gdens}\\ \text{Fdist}\\ \text{Ffreq} \end{array}\right\}\times \text{Sex}+\text{DecTime}+{\text{DecTime}}^{2}}}+\overset{\mathrm{Random}\;\mathrm{component}}{\overbrace{\left(1|\text{BirdID})+(\text{CorStructTp}\;|\text{BirdID}\right)}}$

Across the three analyses, the *Gdens*, *Fdist* and *Ffreq* front metrics were tested for in separate models to avoid issues pertaining from multicollinearity. Sex was included as a fixed factor and in a two-way interaction with each front metric to account for sexual segregation in gannet foraging behaviour [47,53,61]. Time of day (*DecTime*) was included in the dive shape, depth and duration models as a continuous quadratic function to allow for potential changes in the profile of a dive in the middle of the day, possibly owing to the diel migration of prey above and below the thermocline [38,62]. In all models, a random intercept of bird was included to avoid pseudo-replication and account for any individual differences in dive behaviour [6]. Where appropriate, a nested autocorrelation structure was also incorporated to allow for similarities between dives that occurred in temporal clusters. In the habitat-use availability analysis, this was fitted as a spatial correlation structure, using the coordinates of each dive or pseudo-absence location on a universal transverse Mercator projection, whilst for the dive shape, depth and duration analyses, a continuous time correlation structure was used (table 1).

As GLMMs from the MASS package are estimated using penalized quasi-likelihood, maximum-likelihood (ML) selection techniques (e.g. Akaike's information criteria, AIC) were not available, and so the best temporal correlation structure (e.g. exponential, rational quadratic, autoregressive) for the random component (table 1) of the model was selected through inspection of residual plots. Model reduction of the fixed component (table 1) was performed by removing variables with small parameter estimates, relatively large standard errors, confidence intervals that passed through zero and large *p*-values [63–65].

For each LMM, the most appropriate random structure (table 1) was determined via restricted maximum-likelihood (REML) estimation [57] and selected by a comparison of AIC values and residual plots. Model selection of the fixed effects (table 1) was conducted by backwards and forwards selection via ML estimation and the use of AIC and likelihood ratio tests. The most parsimonious model was then refitted using REML to obtain parameter estimates and associated *p*-values [57].

Models were evaluated by plotting Pearson (GLMM) or normalized (LMM) residuals against all potential explanatory variables, bird ID, distance to colony, latitude, longitude, tag type, time and year to check for any patterns indicative of a violation of model assumptions. Fitted versus predicted values were inspected to check for satisfactory model fit, and for the binomial GLMMs the area under the receiving operator characteristic curve (AUC; [66,67]) was calculated. Pseudo-*R*^2^-values were generated as an indication of variance explained [68], using the MuMIn package in R [69]. All analyses were performed in R v. 3.0.2 (R Development Core Team 2013) and Matlab R2011b.

## 3. Results

### 3.1. Gannet tracking data

All 53 birds equipped with GPS and TDR loggers yielded useable data: 11 males and 11 females in 2012 and 17 females and 14 males in 2013. This produced a total of 74 complete and 12 partial foraging trips (figure 1). The number of complete foraging trips per bird averaged 1.4 ± 0.08 (range 1–4).

Maximum displacement (the furthest distance from the colony) averaged 138.5 ± 8.0 km (range 34.6–276.7 km), trip lengths averaged 424.0 ± 25.8 km (range 72.0–822.9 km) and trip duration averaged 23.2 ± 1.5 h (range 2.7–50.6 h).

### 3.2. Overview of gannet diving behaviour

The total number of dives made across all foraging trips was 1901 (figure 1); 712 and 1189 of these were made by males and females, respectively. The number of dives made per complete foraging trip averaged 22.2 ± 3.8, although 17.6% of these trips had no dives. All incomplete foraging trips included dive events. Six birds did not dive at all during deployments.

Dives tended to occur in short bursts rather than being spread out equally over the foraging trip. 25.1%, 39.9% and 58.2% of all dives were followed by another dive within 5, 10 and 20 min respectively, and only 22.9% of dives were spaced more than 1 h from the previous dive. Across complete foraging trips, the number of dives made per hour ranged from 0 to 5.5 with an average of 0.9 ± 0.13. The percentage of time spent underwater during a complete foraging trip was low and ranged from 0% to 0.7% with an average of 0.1 ± 0.02%.

### 3.3. Dive behaviour at fronts

#### 3.3.1. Dive events

**Figure 3.**
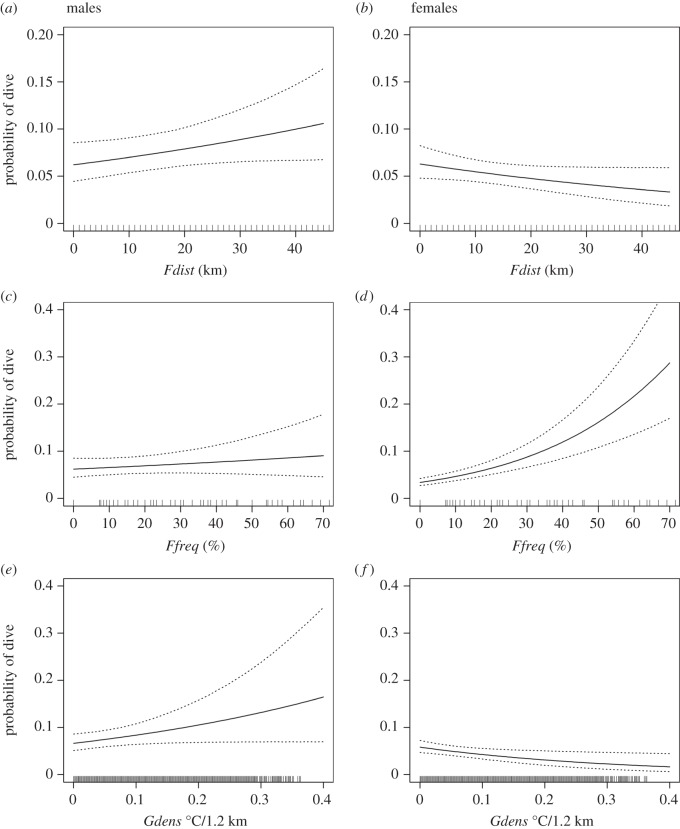
Habitat usage as indicated by the probability of a dive event occurring within a specific habitat type relative to that available. Rows from top to bottom: (*a*,*b*) *Fdist*, (*c*,*d*) *Ffreq* and (*e*,*f*) *Gdens*. The first column shows habitat preferences for males (*a*,*c*,*e*) and the second column for females (*b*,*d*,*f*). Filled line shows expected dive probabilities for an ‘average’ bird. Dotted lines show bootstrapped 95% confidence intervals. Note the change in y-axis extent between the top row and bottom two rows.

Gannets dived in a wide range of oceanographic conditions including areas of high frontal activity (figure 1). When compared with the available habitat types, dive distributions were significantly related to all three of the front metrics (*Fdist*, *Ffreq* and *Gdens*), although relationships varied by sex (figure 3 and table 2). Male dive probabilities were positively correlated with distance to front (*Fdist*), cross-front gradient strength (*Gdens*) and (weakly) with seasonal front frequency (*Ffreq*). Female dive probabilities were negatively correlated with distance to front (*Fdist*) and cross-front gradient strength (*Gdens*), and positively correlated with seasonal front frequency (*Ffreq*; figure 3 and table 2).

**Table 2.** Parameter estimates, standard errors, lower (2.5%) and upper (97.5%) 95% confidence limits and *p*-values for the generalized linear mixed effects model fitted with a random intercept of *BirdID*, nested spatial correlation structure and binomial complementary log–log (cloglog) link function for the habitat usage models. Models fitted, from top to bottom, for: (*a*) *Gdens*, (*b*) *Fdist* and (*c*) *Ffreq*. Base level of the two-state factor for *Sex* is male. The calculated area under the receiver operating characteristic curve (AUC) is indicated. Pseudo-*R*^2^ estimates are quoted as an indication of the variance explained by the fixed component of the model.

fixed effect	estimate	s.e.	lower CI	upper CI	*p*-value	pseudo-*R*^2^ (%)
(*a*) *Gdens*: AUC = 0.53						
* Intercept*	−2.675	0.137	−2.944	−2.407	<0.001	—
* Sex* (*female*)	−0.133	0.178	−0.492	0.226	0.460	—
* Gdens*	2.402	1.273	−0.093	4.897	0.059	—
* Sex* (*female*) * *Gdens*	−5.593	1.819	−9.159	−2.028	0.002	5.0
(*b*) *Fdist*: AUC = 0.56						
* intercept*	−2.745	0.171	−3.080	−2.411	<0.001	—
* Sex* (*female*)	0.014	0.223	−0.436	0.464	0.949	—
* Fdist*	0.012	0.007	−0.001	0.026	0.077	—
* Sex* (*female*) * *Fdist*	−0.027	0.011	−0.048	−0.006	0.013	3.37
(*c*) Ffreq: AUC = 0.55						
* intercept*	−2.745	0.167	−3.070	−2.417	<0.001	—
* Sex* (*female*)	−0.620	0.203	−1.029	−0.212	0.004	—
* Ffreq*	0.006	0.006	−0.007	0.018	0.366	—
* Sex* (*female*) * *Ffreq*	0.027	0.008	0.012	0.042	<0.001	11.5

#### 3.3.2. Dive shape

**Figure 4.**
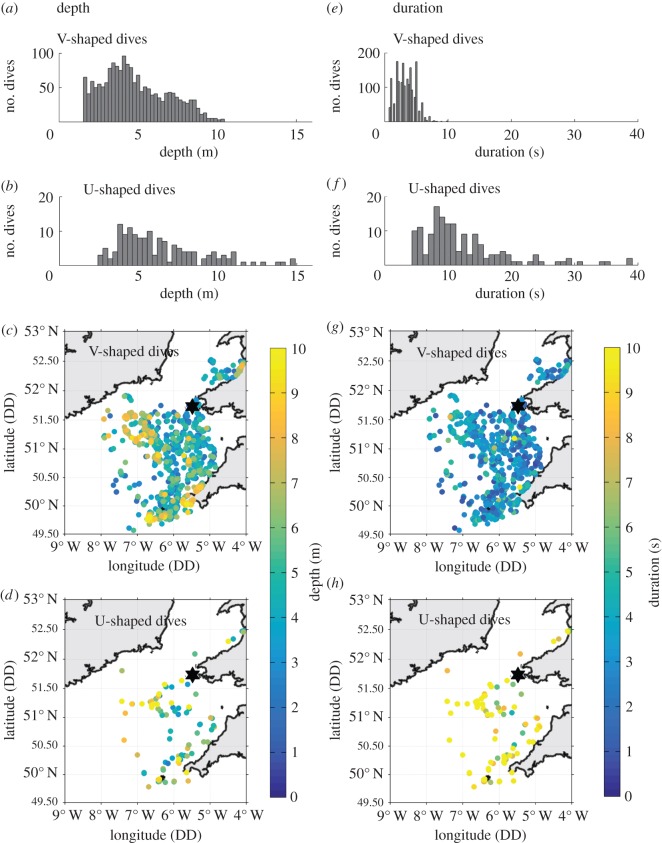
Distributions of gannet dive depths and durations during 2012 and 2013 combined. The left column, from top to bottom shows: frequency distribution of (*a*) V-shaped dive depths and (*b*) U-shaped dive depths, (*c*) the distribution of V-shaped dive depths across the Celtic Sea and (*d*) the distribution of U-shaped dive depths across the Celtic Sea. The right column, from top to bottom shows: frequency distribution of (*e*) V-shaped dive durations and (*f*) U-shaped dive durations, (*g*) the distribution of V-shaped dive durations across the Celtic Sea and (*h*) the distribution of U-shaped dive durations across the Celtic Sea. The location of Grassholm corresponds to the black star on (*c*,*d*,*g*,*h*).

**Figure 5.**
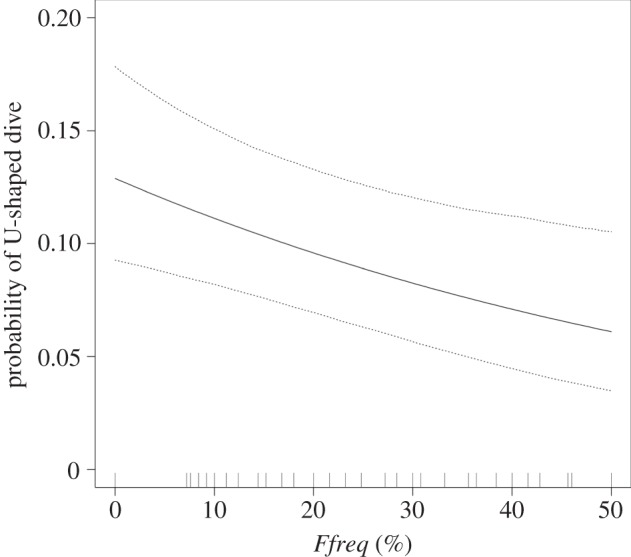
Probability of a U-shaped dive decreases in regions of enhanced frontal activity (*Ffreq*). Filled line shows expected dive shape probabilities for an ‘average’ bird. Dotted lines show bootstrapped 95% confidence intervals.

V-shaped (92.1% of dives) dives were more common than U-shaped dives (7.9% of dives; figure 4). All birds performed V-shaped dives and 69.6% performed U-shaped dives. Dive shape varied significantly with front frequency (*Ffreq*; figure 5 and table 3). When diving in areas of high front frequency, the probability that a bird performed a U-shaped dive compared with a V-shaped dive halved (from approx. 0.12 to 0.06). Dive shape did not change in response to *DecTime*, any of the other front metrics (*Fdist* and *Gdens*), sex or an interaction between sex and frontal activity.

**Table 3.** Parameter estimates, standard errors, lower (2.5%) and upper (97.5%) 95% confidence limits and *p*-values for the generalized linear mixed effects model fitted with a random intercept of *BirdID*, nested temporal correlation structure and binomial complementary log–log (cloglog) link function for dive shape. The area under the receiver operating characteristic curve (AUC) was calculated as 0.8. Pseudo-*R*^2^ estimates are quoted as an indication of the variance explained by the fixed component of the model.

fixed effect	estimate	s.e.	lower CI	upper CI	*p*-value	pseudo-*R*^2^ (%)
*Intercept*	−1.981	0.180	−2.334	−1.627	<0.001	—
*Ffreq*	−0.016	0.006	−0.028	−0.004	0.011	3.3

#### 3.3.3. Dive depth

Overall dive depths ranged from 1.6 to 14.9 m and were greater when birds performed U-shaped compared with V-shaped dives (figure 4). V- and U-shaped dive depths were not significantly related to any of the front metrics. Females consistently dived deeper than males when performing V-shaped dives (4.6 ± 0.4 versus 3.4 ± 0.3 m respectively; *p* = 0.003). U-shaped dive depths averaged 6.3 ± 0.3 m and did not differ between sexes. *DecTime* had no influence on dive depth for either strategy, and there was no significant effect of an interaction between sex and frontal activity.

#### 3.3.4. Dive duration

**Figure 6.**
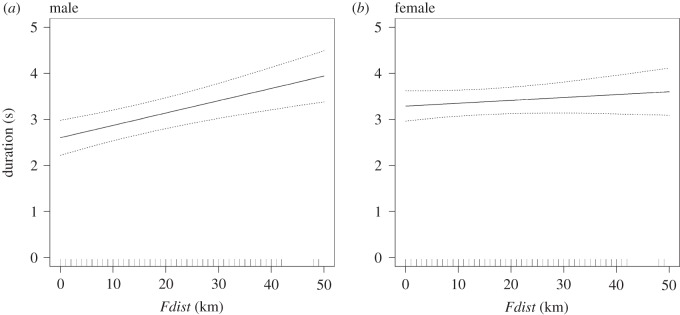
Predicted durations of V-shaped dives at varying proximity to fronts (*Fdist*). From left to right, (*a*) V-shaped dive duration of males increases with distance to nearest front and (*b*) V-shaped dive duration of females increases with distance to nearest front, but not as markedly as observed in males. Filled line shows expected dive durations for an ‘average’ bird. Dotted lines show bootstrapped 95% confidence intervals.

Overall dive durations ranged from 0.7 to 39.1 s and were greater when birds engaged in U-shaped compared with V-shaped dives (figure 4). V-shaped dives were significantly shorter in proximity to fronts (*Fdist*; table 4 and figure 6). This response was more prominent in males (table 4 and figure 6), who had significantly shorter dives than females (table 4). V-shaped dive duration was not significantly related to any of the other front metrics (*Ffreq* and *Gdens*). V-shaped dives were longer in the middle of the day (*DecTime*; table 4 and electronic supplementary material, figure S3). U-shaped dive duration averaged 12.9 ± 0.8 s and did not vary in response to *DecTime*, any of the front metrics, sex or an interaction between sex and frontal activity.

**Table 4.** Parameter estimates, standard errors, lower (2.5%) and upper (97.5%) 95% confidence limits and *p*-values for the linear mixed effects model fitted with a random intercept of *BirdID* for dive duration. Base level of the two-state factor for *Sex* is male. Pseudo-*R*^2^ estimates are quoted as an indication of the variance explained by each explanatory variable within the fixed component of the model. These were generated as the difference in pseudo-*R*^2^ values of models with and without a specific term.

fixed effect	estimate	s.e.	lower CI	upper CI	*p*-value	pseudo*-R*^2^ (%)
*Intercept*	1.468	0.348	0.785	2.152	<0.001	—
*Sex* (*female*)	0.686	0.236	0.210	1.161	0.006	—
*Fdist*	0.027	0.007	0.014	0.040	<0.001	—
*DecTime*	3.754	1.317	1.171	6.337	0.004	—
*DecTime^2^*	−2.965	1.232	−5.381	−0.549	0.016	1.2
*Sex* (*female*) * *Fdist*	−0.021	0.009	−0.038	−0.003	0.020	2.8

## 4. Discussion

This study provides novel insights into the influence of physical oceanography on habitat use and dive behaviour by a medium-ranging piscivorous predator. We build upon prior observations that gannets intensify restricted search behaviours in areas with seasonally persistent fronts [35] to show that these habitats are also favoured for diving, although there was variation between the sexes. In addition, we show that when diving around fronts, gannets are half as likely to engage in U-shaped compared with V-shaped dives and the average duration of V-shaped dives is significantly shortened, which was independent of sex. Combined, these findings are of particular significance because, as well as confirming the importance of shelf-sea fronts as foraging habitat, differences in diving behaviour provide a possible functional mechanism underpinning the links between large marine predators and these physical features.

### 4.1. Persistent fronts as predictable foraging habitats

Our analysis of gannet dive distributions showed females and, to a lesser extent, males, preferentially dived in regions of persistent frontal activity. This adds to a growing body of evidence documenting the importance of such features as foraging habitats for marine predators [21,34,35,70–72]. However, patterns were far less clear around shorter-term, more ephemeral fronts. Here, male dive probabilities were positively correlated with distance to front (*Fdist*) and cross-front gradient strength (*Gdens*), with the reverse for females. Across shelf-seas, persistence is a key feature of the bioaggregating fronts [8] that are associated with high levels of primary productivity and biomass accumulation which sustains low- to mid-trophic-level enhancement and increases prey abundance (e.g. tidal-mixing fronts; [16,20,21,73]). Moreover, dependent upon spatio-temporal scale [74,75], these features may occur in a highly predictable manner [34], which likely aids individuals in efficiently locating their prey through learning, knowledge transfer and/or memory [4,76–80]. The significance of small ephemeral features (*Gdens*) therefore requires further study.

### 4.2. Subsurface dive behaviour around fronts

The principal purpose of this study was to investigate the subsurface movements of gannets in relation to shelf-sea fronts, to better understand their functional significance. In addition to providing persistent and predictable foraging habitats, fronts are also thought to increase the catchability and accessibility of prey [20,22]. In gannets, foraging strategies are especially energetically expensive [48,52,81,82], and to maximize efficiency individuals adjust their underwater movements in response to the behaviours and depth distributions of their prey [49,51,83,84]. V-shaped dives dominated gannet foraging strategies across the Celtic Sea, which possibly suggests this method of prey capture is better suited than a U-shaped dive strategy to the types of prey naturally encountered in the region (e.g. fast swimming pelagic species; [39,49,50]). We hypothesize that the shorter duration and more frequent use of passive V-shaped dives (i.e. with little or no active swim phase) around fronts is related to improvements in the availability of this prey.

Strong biophysical coupling at fronts is thought to attract large numbers of the mid-trophic-level fish (e.g. garfish, herring and mackerel) that gannets feed on [39]. Resultant high prey densities [22] may increase encounter probabilities [85] which aid in capture, allowing for faster and shorter dives [37,48]. Moreover, pursuit dives (U-shaped) are thought to be less suitable for catching highly responsive shoaling fish [86], which instead are better ambushed during fast V-shaped dives [50]. Fronts may also concentrate fish close to the surface, making them easier for gannets to catch. This is due to increased primary productivity and biomass accumulation around the near-surface thermocline [16,20,73], and because some fish may actively avoid cool bottom-boundary layer waters (e.g. mackerel; [50,87]). V-shaped dives were shallower than U-shaped dives (3.43/4.62 m for males and females respectively versus 6.32 m) and so an increase in their use around fronts may reflect this shallow distribution of fish [37,51]. In some instances, gannets may additionally cue in on other marine predators such as cetaceans [88–90] or other seabirds [90] that also forage around fronts [71,91,92]. Under such instances, mixed-species foraging aggregations may increase foraging success through the disorganization of school cohesiveness [93], or by preventing prey escaping to deeper waters when under attack [94].

### 4.3. Identifying important habitat features for foraging marine predators in dynamic ecological systems

There was a low signal-to-noise ratio across all of our analyses, and as such, the resultant variances explained were low (less than 12%) whilst model fits were sometimes poor (AUC of use-availability models less than 0.6; [66]). This was likely to be a by-product of the highly dynamic nature of shelf-sea environments coupled to the multiple trophic connections required to link physical features to gannets. However, despite this complexity, we were able to identify biologically plausible mechanisms at play, consistent with our *a priori* predictions about the importance of fronts as foraging habitat. Composite front mapping techniques probably played an important role in this by objectively defining and identifying frontal features across multiple spatio-temporal scales, which allowed transient ephemeral features (*Gdens*) to be separated from the more persistent and predictable fronts used by gannets (*Ffreq*).

In some instances, suitable habitat locations may be unused owing to mechanisms other than those being investigated. For example, frequent frontal zones around the coast of Ireland (figure 1) were probably avoided as a result of competition with neighbouring colonies and resultant space segregation [95], whilst those occurring around the mouth of the Bristol Channel are possibly associated with waters whose turbidity hinders the foraging ability of a visual forager such as the gannet [96]. The use of a habitat-use availability analysis, that included only areas deemed accessible for foraging as defined by kernel density analyses of GPS tracks, minimized the influence of these potentially interfering processes.

Individual and sex-specific foraging specialization may further obfuscate relationships between gannet dive behaviours and physical oceanography [6,40,41,53,61]. Correlations between the dive distributions of males and frequent frontal zones (*Ffreq*) were weaker than those observed with females (figure 3*c*,*d*). Moreover, there were contrasting negative and positive relationships with distance to front (*Fdist*; figure 3*a*,*b*). Sex-specific differences in habitat use by gannets have also been observed in the North Sea, where males preferentially forage in near-shore coastal regions, whilst females target offshore areas of intermediate SSTs where fronts would ordinarily manifest [53]. The underlying mechanisms driving sex-specific differences in front use described here are unclear, but could be related to contrasting parental roles [97], interference competition [98], habitat segregation [53] and/or differences in nutritional requirements/prey preference [61]. As such, we highlight the need for a comprehensive knowledge of inter- and intraspecies-specific behaviours when investigating the drivers of marine predator habitat selection [6,95,99].

As well as feeding naturally on pelagic fishes, gannets also feed on discards from commercial fisheries [40,41,61], and scavenging may erode the relationships between diving gannets and fronts. To test this, we re-modelled habitat-use availability excluding all dives associated with fishing vessels (length > 15 m) and their pseudo-absences, and re-analysed changes in dive duration and shape around fronts including presence/absence of fishing vessels as a two-level factor. Dives were assumed to be at a fishing boat when within 10 km and 1 h of a logged vessel location (17.6% and 20.9% of female and male dives, respectively, as indicated by the UK vessel monitoring system provided by the Centre for Environment, Fisheries and Aquaculture Science; [39,41,100]). We found that across these re-analyses, model outputs were consistent with previous investigations that did not account for/exclude dives associated with fishing boats (for dive duration and shape re-analyses, the factor variable of fishing boat presence was not retained following model reduction). As such, whilst scavenging waste from fisheries may increase noise in this system (the influence of boats under 15 m could not be ascertained), there is no evidence that this systematically altered our findings, which is perhaps an indication of the strength of association between gannets and fronts in the Celtic Sea.

## 5. Conclusion

It has been suggested that shelf-sea fronts constitute key components in the functioning of marine ecosystems by providing marine predators with persistent, predictable and productive foraging habitats [8,20,21]. Our work reiterates this assertion and highlights the key role these features play in shaping both the distributions and foraging behaviours of gannets. Moreover, we suggest an increase in the use of short V-shaped dive strategies around fronts reflects improved prey accessibility and catchability. Our study highlights the complexities of interactions between marine vertebrate predators and their environment, and the subsequent importance of collaboration between disciplines (spatial ecology, oceanography and remote-sensing). We show that studies combining fine-scale foraging behaviours and remotely sensed measurements of physical oceanography can provide valuable insights towards the mechanisms that drive the at-sea distributions of marine predators. As such, there is a pressing need for cross-disciplinary research when attempting to understand marine vertebrate ecology and how marine ecosystems function.

## Supplementary Material

Supplementary material

## Supplementary Material

Supporting data for habitat use-availability analyses

## Supplementary Material

Supporting data for dive behaviour analyses

## Data accessibility

Supporting datasets can be found in the electronic supplementary material.

## References

[1] Weimerskirch H, Doncaster CP, Cuenot-Chaillet F 1994 Pelagic seabirds and the marine environment: foraging patterns of wandering albatrosses in relation to prey availability and distribution. Proc. R. Soc. Lond. B 255, 91–97. (doi:10.1098/rspb.1994.0013)

[2] Sims DW et al. 2008 Scaling laws of marine predator search behaviour. Nature 451, 1098–1102. (doi:10.1038/nature06518) 18305542 10.1038/nature06518

[3] Pinaud D, Weimerskirch H 2005 Scale-dependent habitat use in a long-ranging central place predator. J. Anim. Ecol. 74, 852–863. (doi:10.1111/j.1365-2656.2005.00984.x)

[4] Weimerskirch H 2007 Are seabirds foraging for unpredictable resources? Deep-Sea Res. Part II 54, 211–223. (doi:10.1016/j.dsr2.2006.11.013)

[5] Hamer KC, Humphreys EM, Magalhães MC, Garthe S, Hennicke J, Peters G, Grémillet D, Skov H, Wanless S 2009 Fine-scale foraging behaviour of a medium-ranging marine predator. J. Anim. Ecol. 78, 880–889. (doi:10.1111/j.1365-2656.2009.01549.x) 19426254 10.1111/j.1365-2656.2009.01549.x

[6] Patrick SC et al. 2014 Individual differences in searching behaviour and spatial foraging consistency in a central place marine predator. Oikos 123, 33–40. (doi:10.1111/j.1600-0706.2013.00406.x)

[7] Bost CA, Cotté C, Bailleul F, Cherel Y, Charrassin JB, Guinet C, Ainley DG, Weimerskirch H 2009 The importance of oceanographic fronts to marine birds and mammals of the southern oceans. J. Mar. Syst. 78, 363–376. (doi:10.1016/j.jmarsys.2008.11.022)

[8] Scales KL, Miller PI, Hawkes LA, Ingram SN, Sims DW, Votier SC 2014 On the front line: frontal zones as priority at-sea conservation areas for mobile marine vertebrates. J. Appl. Ecol. 51, 1575–1583. (doi:10.1111/1365-2664.12330)

[9] De Monte S, Cotté C, d'Ovidio F, Lévy M, Le Corre M, Weimerskirch H 2012 Frigatebird behaviour at the ocean–atmosphere interface: integrating animal behaviour with multi-satellite data. J. R. Soc. Interface. 9, 3351–3358. (doi:10.1098/rsif.2012.0509) 22951344 10.1098/rsif.2012.0509 PMC3481590

[10] Cotté C, d'Ovidio F, Chaigneau A, Lévy M, Taupier-Letage I, Mate B, Guinet C 2011 Scale-dependent interactions of Mediterranean whales with marine dynamics. Limnol. Oceanogr. 56, 219–232. (doi:10.4319/lo.2011.56.1.0219)

[11] Benjamins S, Dale A, Hastie G, Waggitt J, Lea MA, Scott B, Wilson B 2015 Confusion reigns? A review of marine megafauna interactions with tidal-stream environments. Oceanogr. Mar. Biol. Annu. Rev. 53, 1–54. (doi:10.1201/b18733-2)

[12] Scott BE, Sharples J, Ross ON, Wang J, Pierce GJ, Camphuysen CJ 2010 Sub-surface hotspots in shallow seas: fine-scale limited locations of top predator foraging habitat indicated by tidal mixing and sub-surface chlorophyll. Mar. Ecol. Prog. Ser. 408, 207–226. (doi:10.3354/meps08552)

[13] Embling CB, Illian J, Armstrong E, van der Kooij J, Sharples J, Camphuysen KCJ, Scott BE 2012 Investigating fine-scale spatio-temporal predator-prey patterns in dynamic marine ecosystems: a functional data analysis approach. J. Appl. Ecol. 49, 481–492. (doi:10.1111/j.1365-2664.2012.02114.x)

[14] Embling CB, Sharples J, Armstrong E, Palmer MR, Scott BE 2013 Fish behaviour in response to tidal variability and internal waves over a shelf sea bank. Prog. Oceanogr. 117, 106–117. (doi:10.1016/j.pocean.2013.06.013)

[15] Simpson JH, Hunter JR 1974 Fronts in the Irish Sea. Nature 250, 404–406. (doi:10.1038/250404a0)

[16] Franks PJS 1992 Phytoplankton blooms at fronts: patterns, scales, and physical forcing mechanisms. Rev. Aquat. Sci. 6, 121–137.

[17] Yoder JA, Ackleson SG, Barber RT, Flament P, Balch WM 1994 A line in the sea. Nature 371, 689–692. (doi:10.1038/371689a0)

[18] Genin A, Jaffe JS, Reef R, Richter C, Franks PJS 2005 Swimming against the flow: a mechanism of zooplankton aggregation. Science 308, 860–862. (doi:10.1126/science.1107834) 15879218 10.1126/science.1107834

[19] Decker MB, Hunt GL 1996 Foraging by murres (*Uria* spp.) at tidal fronts surrounding the Pribilof Islands, Alaska, USA. Mar. Ecol. Prog. Ser. 139, 1–10. (doi:10.3354/meps139001)

[20] Russell RW, Harrison NM, Hunt GL 1999 Foraging at a front: hydrography, zooplankton, and avian planktivory in the northern Bering Sea. Mar. Ecol. Prog. Ser. 182, 77–93. (doi:10.3354/meps182077)

[21] Jahncke J, Coyle KO, Zeeman SI, Kachel NB, Hunt GL 2005 Distribution of foraging shearwaters relative to inner front of SE Bering Sea. Mar. Ecol. Prog. Ser. 305, 219–233. (doi:10.3354/meps305219)

[22] Vlietstra LS, Coyle KO, Kachel NB, Hunt GL 2005 Tidal front affects the size of prey used by a top marine predator, the short-tailed shearwater (*Puffinus tenuirostris*). Fish Oceanogr. 14, 196–211. (doi:10.1111/j.1365-2419.2005.00369.x)

[23] Ainley DG, Ribic CA, Woehler EJ 2012 Adding the ocean to the study of seabirds: a brief history of at-sea seabird research. Mar. Ecol. Prog. Ser. 451, 231–243. (doi:10.3354/meps09524)

[24] Wakefield ED, Phillips RA, Matthiopoulos J 2009 Quantifying habitat use and preferences of pelagic seabirds using individual movement data: a review. Mar. Ecol. Prog. Ser. 391, 165–182. (doi:10.3354/meps08203)

[25] Elliott KH, Woo K, Gaston AJ, Benvenuti S, Dall'Antonia L, Davoren GK 2008 Seabird foraging behaviour indicates prey type. Mar. Ecol. Prog. Ser. 354, 289–303. (doi:10.3354/meps07221)

[26] Boyd C, Castillo R, Hunt GL, Punt AE, VanBlaricom GR, Weimerskirch H, Bertrand S 2015 Predictive modelling of habitat selection by marine predators with respect to the abundance and depth distribution of pelagic prey. J. Anim. Ecol. 84, 1575–1588. (doi:10.1111/1365-2656.12409) 26061120 10.1111/1365-2656.12409

[27] Goldbogen JA, Hazen EL, Friedlaender AS, Calambokidis J, DeRuiter SL, Stimpert AK, Southall BL 2015 Prey density and distribution drive the three-dimensional foraging strategies of the largest filter feeder. Funct. Ecol. 29, 951–961. (doi:10.1111/1365-2435.12395)

[28] Bograd SJ, Block BA, Costa DP, Godley BJ 2010 Biologging technologies: new tools for conservation. Introduction. Endanger Species Res. 10, 1–7. (doi:10.3354/esr00269)

[29] Burger AE 2003 Effects of the Juan de Fuca Eddy and upwelling on densities and distributions of seabirds off southwest Vancouver Island, British Columbia. Mar. Ornithol. 31, 113–122.

[30] Gremillet D et al. 2008 Spatial match–mismatch in the Benguela upwelling zone: should we expect chlorophyll and sea-surface temperature to predict marine predator distributions. J. Appl. Ecol. 45, 610–621. (doi:10.1111/j.1365-2664.2007.01447.x)

[31] Miller P 2009 Composite front maps for improved visibility of dynamic sea-surface features on cloudy SeaWiFS and AVHRR data. J. Mar. Syst. 78, 327–336. (doi:10.1016/j.jmarsys.2008.11.019)

[32] Miller PI, Christodoulou S 2014 Frequent locations of oceanic fronts as an indicator of pelagic diversity: application to marine protected areas and renewables. Mar. Policy 45, 318–329. (doi:10.1016/j.marpol.2013.09.009)

[33] Scales KL, Miller PI, Varo-Cruz N, Hodgson DJ, Hawkes LA, Godley BJ 2015 Oceanic loggerhead turtles *Caretta caretta* associate with thermal fronts: evidence from the Canary Current Large Marine Ecosystem. Mar. Ecol. Prog. Ser. 519, 195–207. (doi:10.3354/meps11075)

[34] Miller PI, Scales KL, Ingram SN, Southall EJ, Sims DW 2015 Basking sharks and oceanographic fronts: quantifying associations in the north-east Atlantic. Funct. Ecol. 29, 1099–1109. (doi:10.1111/1365-2435.12423)

[35] Scales KL, Miller PI, Embling CB, Ingram SN, Pirotta E, Votier SC 2014 Mesoscale fronts as foraging habitats: composite front mapping reveals oceanographic drivers of habitat use for a pelagic seabird. J. R. Soc. Interface 11, 20140679 (doi:10.1098/rsif.2014.0679) 25165595 10.1098/rsif.2014.0679 PMC4191095

[36] Hamer KC, Humphreys EM, Garthe S, Hennicke J, Peters G, Grémillet D, Phillips RA, Harris MP, Wanless S 2007 Annual variation in diets, feeding locations and foraging behaviour of gannets in the North Sea: flexibility, consistency and constraint. Mar. Ecol. Prog. Ser. 338, 295–305. (doi:10.3354/meps338295)

[37] Ropert-Coudert Y, Daunt F, Kato A, Ryan PG, Lewis S, Kobayashi K, Mori Y, Grémillet D, Wanless S 2009 Underwater wingbeats extend depth and duration of plunge dives in northern gannets *Morus bassanus*. J. Avian Biol. 40, 380–387. (doi:10.1111/j.1600-048X.2008.04592.x)

[38] Garthe S, Benvenuti S, Montevecchi WA 2000 Pursuit plunging by northern gannets (*Sula bassana*) feeding on capelin (*Mallotus villosus*). Proc. R. Soc. Lond. B 267, 1717–1722. (doi:10.1098/rspb.2000.1200) 10.1098/rspb.2000.1200 PMC1690745 12233767

[39] Votier SC, Bearhop S, Witt MJ, Inger R, Thompson D, Newton J 2010 Individual responses of seabirds to commercial fisheries revealed using GPS tracking, stable isotopes and vessel monitoring systems. J. Appl. Ecol. 47, 487–497. (doi:10.1111/j.1365-2664.2010.01790.x)

[40] Votier SC, Bicknell A, Cox SL, Scales KL, Patrick S 2013 A bird's eye view of discard reforms: bird-borne cameras reveal seabird/fishery interactions. PLoS ONE 8, e57376 (doi:10.1371/journal.pone.0057376) 23483906 10.1371/journal.pone.0057376 PMC3590202

[41] Patrick SC, Bearhop S, Bodey TW, Grecian WJ, Hamer KC, Lee J, Votier SC 2015 Inidividual seabirds show consistent foraging strategies in response to predictable fisheries discards. J. Avian Biol. 46, 431–440. (doi:10.1111/jav.00660)

[42] Sabarros PS, Grémillet D, Demarcq H, Moseley C, Pichegru L, Mullers RHE, Stenseth NC, Machu E 2014 Fine-scale recognition and use of mesoscale fronts by foraging Cape gannets in the Benguela upwelling region. Deep-Sea Res. II 107, 77–84. (doi:10.1016/j.dsr2.2013.06.023)

[43] Vazquez-Prokopec GM, Stoddard ST, Paz-Soldan V, Morrison AC, Elder JP, Kochel TJ, Scott TW, Kitron U 2009 Usefulness of commercially available GPS data-loggers for tracking human movement and exposure to dengue virus. Int. J. Health Geogr. 8, 68 (doi:10.1186/1476-072X-8-68) 19948034 10.1186/1476-072X-8-68 PMC2792221

[44] Garthe S, Grémillet D, Furness RW 1999 At-sea-activity and foraging efficiency in chick-rearing northern gannets *Sula bassana*: a case study in Shetland. Mar. Ecol. Prog. Ser. 185, 93–99. (doi:10.3354/meps185093)

[45] Ropert-Coudert Y, Gremillet D, Kato A, Ryan PG, Naito Y, Le Maho Y 2004 A fine-scale time budget of Cape gannets provides insights into the foraging strategies of coastal seabirds. Anim. Behav. 67, 985–992. (doi:10.1016/j.anbehav.2003.09.010)

[46] Carter MID et al. 2015 GPS tracking reveals rafting behaviour of northern gannets (*Morus bassanus*): implications for foraging ecology and conservation. Bird Study J. Br. Trust Ornithol. 63, 83–95. (doi:10.1080/00063657.2015.1134441)

[47] Lewis S, Benvenuti S, Dall-Antonia L, Griffiths R, Money L, Sherratt TN, Wanless S, Hamer KC 2002 Sex-specific foraging behaviour in a monomorphic seabird. Proc. R. Soc. B 269, 1687–1693. (doi:10.1098/rspb.2002.2083) 10.1098/rspb.2002.2083 PMC1691079 12204129

[48] Ropert-Coudert Y, Gremillet D, Ryan P, Kato A, Naito Y, Le Maho Y 2004 Between air and water: the plunge dive of the Cape gannet *Morus capensis*. Ibis 146, 281–290. (doi:10.1111/j.1474-919x.2003.00250.x)

[49] Garthe S, Montevecchi WA, Chapdelaine G, Rail JF, Hedd A 2007 Contrasting foraging tactics by northern gannets (*Sula bassana*) breeding in different oceanographic domains with different prey fields. Mar. Biol. 151, 687–694. (doi:10.1007/s00227-006-0523-x)

[50] Garthe S, Guse N, Montevecchi WA, Rail J-F, Grégoire F 2014 The daily catch: flight altitude and diving behaviour of northern gannets feeding on Atlantic mackerel. J. Sea Res. 85, 456–462. (doi:10.1016/j.seares.2013.07.020)

[51] Machovsky-Capuska GE, Vaughn RL, Wursig B, Katzir G, Raubenheimer D 2011 Dive strategies and foraging effort in the Australasian gannets *Morus serrator* revealed by underwater videography. Mar. Ecol. Prog. Ser. 442, 255–261. (doi:10.3354/meps09458)

[52] Green JA, White CR, Bunce A, Frappell PB, Butler PJ 2010 Energetic consequences of plunge diving in gannets. Endanger Species Res. 10, 269–279. (doi:10.3354/esr00223)

[53] Cleasby IR, Wakefield ED, Bodey TW, Davies RD, Patrick SC, Newton J, Votier SC, Bearhop S, Hamer KC 2015 Sexual segregation in a wide-ranging marine predator is a consequence of habitat selection. Mar. Ecol. Prog. Ser. 518, 1–12. (doi:10.3354/meps11112)

[54] Belkin IM, Cornillon PC, Sherman K 2009 Fronts in large marine ecosystems. Prog. Oceanogr. 81, 223–236. (doi:10.1016/j.pocean.2009.04.015)

[55] Cayula J-F, Cornillon P 1992 Edge detection algorithm for SST images. J. Atmos. Ocean Technol. 9, 67–80. (doi:10.1175/1520-0426(1992)009<0067:EDAFSI>2.0.CO;2)

[56] Ripley B, Venables B, Bates DM, Hornik K, Gebhardt A, Firth D 2014 MASS (https://cran.r-project.org/web/packages/MASS/MASS.pdf)

[57] Zuur AF, Ieno EN, Walker NJ, Saveliev AA, Smith GM 2009 *Mixed effects models and extensions in ecology with R*. New York, NY: Springer.

[58] Hamel S, Yoccoz NG, Gaillard J-M 2012 Statistical evaluation of parameters estimating autocorrelation and individual heterogeneity in longitudinal studies. Methods Ecol. Evol. 3, 731–742. (doi:10.1111/j.2041-210X.2012.00195.x)

[59] Aarts G, MacKenzie ML, McConnell B, Fedak M, Matthiopoulos J 2008 Estimating space-use and habitat preference from wildlife telemetry data. Ecography 31, 140–160. (doi:10.1111/j.2007.0906-7590.05236.x)

[60] Pinheiro J, Bates DM 2014 nlme: linear and nonlinear mixed effects models (https://cran.r-project.org/web/packages/nlme/nlme.pdf)

[61] Stauss C et al. 2012 Sex-specific foraging behaviour in northern gannets *Morus bassanus*: incidence and implications. Mar. Ecol. Prog. Ser. 457, 151–162. (doi:10.3354/meps09734)

[62] Garthe S, Montevecchi WA, Davoren GK 2007 Flight destinations and foraging behaviour of northern gannets (*Sula bassana*) preying on a small forage fish in a low-Arctic ecosystem. Deep-Sea Res. II 54, 311–320. (doi:10.1016/j.dsr2.2006.11.008)

[63] Bolker BM, Brooks ME, Clark CJ, Geange SW, Poulsen JR, Stevens MHH, White J-SS 2009 Generalized linear mixed models: a practical guide for ecology and evolution. Trends Ecol. Evol. 24, 127–135. (doi:10.1016/j.tree.2008.10.008) 19185386 10.1016/j.tree.2008.10.008

[64] Murtaugh PA 2014 In defence of *P* values. Ecology 95, 611–617. (doi:10.1890/13-0590.1) 24804441 10.1890/13-0590.1

[65] Stanton-Geddes J, Gomes de Freitas C, De Sales Dambros C 2014 In defence of *P* values: comment on the statistical methods actually used by ecologists. Ecology 95, 637–642. (doi:10.1890/13-1156.1) 24804446 10.1890/13-1156.1

[66] Zweig MH, Campbell G 1993 Receiver-operating characteristic (ROC) plots: a fundamental evaluation tool in clinical medicine. Clin Chem. 39, 561–577. 8472349

[67] Liu C, Berry PM, Dawson TP, Pearson RG 2005 Selecting thresholds of occurrence in the prediction of species distributions. Ecography 28, 385–393. (doi:10.1111/j.0906-7590.2005.03957.x)

[68] Nakagawa S, Schielzeth H 2013 A general and simple method for obtaining *R*^2^ from generalized linear mixed-effects models. Methods Ecol. Evol. 4, 133–142. (doi:10.1111/j.2041-210x.2012.00261.x)

[69] Barton K 2014 MuMIn (https://cran.r-project.org/web/packages/MuMIn/MuMIn.pdf)

[70] Durazo R, Harrison NM, Hill AE 1998 Seabird observations at a tidal mixing front in the Irish Sea. Estuar. Coast Shelf Sci. 47, 153–164. (doi:10.1006/ecss.1998.0339)

[71] Doniol-Valcroze T, Berteaux D, Larouche P, Sears R 2007 Influence of thermal fronts on habitat selection by four rorqual whale species in the Gulf of St. Lawrence. Mar. Ecol. Prog. Ser. 335, 207–216. (doi:10.3354/meps335207)

[72] Kokubun N, Iida K, Mukai T 2008 Distribution of murres (*Uria* spp.) and their prey south of St. George Island in the southeastern Bering Sea during the summers of 2003–2005. Deep-Sea Res. II 55, 1827–1836. (doi:10.1016/j.dsr2.2008.04.018)

[73] Franks PJS 1992 Sink or swim: accumulation of biomass at fronts. Mar. Ecol. Prog. Ser. 82, 1–12. (doi:10.3354/meps082001)

[74] Nahas EL, Pattiaratchi CB, Ivey GN 2005 Processes controlling the positions of frontal systems in Shark Bay, Western Australia. Estuar. Coast Shelf Sci. 65, 463–474. (doi:10.1016/j.ecss.2005.06.017)

[75] Pisoni JP, Rivas AK, Piola AR 2015 On the variability of tidal fronts on a macrotidal continental shelf, Northern Patagonia, Argentina. Deep-Sea Res. II 119, 61–68. (doi:10.1016/j.dsr2.2014.01.019)

[76] Pettex E, Bonadonna F, Enstipp MR, Siorat F, Grémillet D 2010 Northern gannets anticipate the spatio-temporal occurrence of their prey. J. Exp. Biol. 213, 2365–2371. (doi:10.1242/jeb.042267) 20581265 10.1242/jeb.042267

[77] Regular PM, Hedd A, Montevecchi WA 2013 Must marine predators always follow scaling laws? Memory guides the foraging decisions of a pursuit-diving seabird. Anim. Behav. 86, 545–552. (doi:10.1016/j.anbehav.2013.06.008)

[78] Machovsky-Capuska GE, Hauber ME, Libby E, Amiot C, Raubenheimer D 2014 The contribution of private and public information in foraging by Australasian gannets. Anim. Cogn. 17, 849–858. (doi:10.1007/s10071-013-0716-x) 24337907 10.1007/s10071-013-0716-x

[79] Wakefield ED, Cleasby IR, Bearhop S, Bodey TW, Davies RD, Miller PI, Newton J, Votier SC, Hamer KC 2015 Long-term individual foraging site fidelity --- why some gannets don't change their spots. Ecology 96, 3058–3074. (doi:10.1890/14-1300.1) 27070024 10.1890/14-1300.1

[80] Davoren GK 2013 Distribution of marine predator hotspots explained by persistent areas of prey. Mar. Biol. 160, 3043–3058. (doi:10.1007/s00227-013-2294-5)

[81] Furness RW, Tasker ML 2000 Seabird-fishery interactions: quantifying the sensitivity of seabirds to reductions in sandeel abundance, and identification of key areas for sensitive seabirds in the North Sea. Mar. Ecol. Prog. Ser. 202, 253–264. (doi:10.3354/meps202253)

[82] Amelineau F, Peron C, Lescroel A, Authier M, Provost P, Gremillet D 2014 Windscape and tortuosity shape the flight costs of northern gannets. J. Exp. Biol. 217, 876–885. (doi:10.1242/jeb.097915) 24622894 10.1242/jeb.097915

[83] Garthe S, Montevecchi WA, Davoren GK 2011 Inter-annual changes in prey fields trigger different foraging tactics in a large marine predator. Limnol. Oceanogr. 56, 802–812. (doi:10.4319/lo.2011.56.3.0802)

[84] Machovsky-Capuska GE, Vaughn RL, Wursig B, Raubenheimer D 2013 Can gannets (*Morus serrator*) select their diving profile prior to submergence? Notornis 60, 255–257.

[85] Enstipp MR, Grémillet D, Jones DR 2007 Investigating the functional link between prey abundance and seabird predatory performance. Mar. Ecol. Prog. Ser. 331, 267–279. (doi:10.3354/meps331267)

[86] Crook KA, Davoren GK 2014 Underwater behaviour of common murres foraging on capelin: influences of prey density and antipredator behaviour. Mar. Ecol. Prog. Ser. 501, 279–290. (doi:10.3354/meps10696)

[87] Grégoire F 2006 Vertical distribution of the midwater trawl catches of Atlantic mackerel (*Scomber scombrus* L.) in relation with water temperature, Research document 2006/097. Fisheries and Oceans Canada (http://www.dfo-mpo.gc.ca/csas-sccs/publications/resdocs-docrech/2006/2006_097-eng.htm)

[88] Camphuysen CJ, Webb A 1999 Multi-species feeding associations in North Sea seabirds: jointly exploiting a patchy environment. Ardea 87, 177–198.

[89] Davoren GK, Garthe S, Montevecchi WA, Benvenuti S 2010 Influence of prey behaviour and other predators on the foraging activities of a marine avian predator in a Low Arctic ecosystem. Mar. Ecol. Prog. Ser. 404, 275–287. (doi:10.3354/meps08370)

[90] Tremblay Y, Thiebault A, Mullers R, Pistorius P 2014 Bird-borne video-cameras show that seabird movement patterns relate to previously unrevealed proximate environment, not prey. PLoS ONE 9, e88424 (doi:10.1371/journal.pone.0088424) 24523892 10.1371/journal.pone.0088424 PMC3921161

[91] Goold JC 1998 Acoustic assessment of populations of common dolphin off the west Wales coast, with perspectives from satellite infrared imagery. J. Mar. Biol. Assoc. UK 78, 1353–1364. (doi:10.1017/S0025315400044544)

[92] Dalla Rosa L, Ford JKB, Trites AW 2012 Distribution and relative abundance of humpback whales in relation to environmental variables in coastal British Columbia waters. Cont. Shelf Res. 36, 89–104. (doi:10.1016/j.csr.2012.01.017)

[93] Thiebault A, Semeria M, Lett C, Tremblay Y 2015 How to capture fish in a school? Effect of successive predator attacks on seabird feeding success. J. Anim. Ecol. 85, 157–167. (doi:10.1111/1365-2656.12455) 26768335 10.1111/1365-2656.12455

[94] Vaughn RL, Wursig B, Shelton DS, Timm LL, Watson LA 2008 Dusky dolphins influence prey accessibility for seabirds in admiralty bay, New Zealand. J. Mammal. 89, 1051–1058. (doi:10.1644/07-MAMM-A-145.1)

[95] Wakefield ED et al. 2013 Space partitioning without territoriality in gannets. Science 341, 68–70. (doi:10.1126/science.1236077) 23744776 10.1126/science.1236077

[96] Machovsky-Capuska GE, Howland HC, Raubenheimer D, Vaughn-Hirshorn R, Wursig B, Hauber ME, Katzir G 2012 Visual accommodation and active pursuit of prey in a plunge-diving bird: the Australasian gannet. Proc. R. Soc. B 279, 4118–4125. (doi:10.1098/rspb.2012.1519) 10.1098/rspb.2012.1519 PMC3441088 22874749

[97] Fraser GS, Jones IL, Hunter FM 2002 Male-female differences in parental care in monogamous crested auklets. The Condor 104, 413–423. (doi:10.1650/0010-5422(2002)104[0413:MFDIPC]2.0.CO;2)

[98] Shealer DA, Burger J 1993 Effects of interference competition on the foraging activity of tropical roseate terns. The Condor 95, 322–329. (doi:10.2307/1369355)

[99] Zavalaga CB, Benvenuti S, Dall'Antonia L, Emslie SD 2007 Diving behaviour of blue-footed boobies *Sula nebouxii* in northern Peru in relation to sex, body size and prey type. Mar. Ecol. Prog. Ser. 336, 291–303. (doi:10.3354/meps336291)

[100] Bodey TW, Jessopp MJ, Votier SC, Gerritsen HD, Cleasby IR, Hamer KC, Patrick SC, Wakefield ED, Bearhop S 2014 Seabird movement reveals the ecological footprint of fishing vessels. Curr. Biol. 24, R514–R515. (doi:10.1016/j.cub.2014.04.041) 24892908 10.1016/j.cub.2014.04.041

