# Supplementary material for: Seabird diving behaviour reveals the functional significance of shelf-sea fronts as foraging hotspots
Source: R Soc Open Sci. 2016 Sep 21;3(9):160317. doi: 10.1098/rsos.160317 (PMC5043317; doi:10.1098/rsos.160317)
Supplement: Supplementary material [file rsos160317supp1.docx]

**Supplementary material**

**S.1 Dive classification methods**

To determine the length of the active swim phase, gradients in the vertical change in depth were used to split dives into three phases; unaided descent (plunge), active swim phase and ascent (Figure S1).

Using the depth data from the CEFAS tags, 100 dive profiles from a random sub-sample were allocated U- or V- shapes based on the length of the active swim phase determined by visual inspection of dive profiles and changes in vertical descent gradient (change in depth/time to give ms^-1^ which was smoothed using a LOWESS smoother with f=0.125). The length of the active swim phase was based on the findings of [1], and started at the end of the plunge phase of a dive and ended at the beginning of the final ascent of the dive. From these 100 sub-sampled dives, descent gradient thresholds were then estimated and used in an algorithm which was then applied across the whole dataset and validated against the manually determined dive shapes of the sub-sample. Additional visual inspection of the defined active swim phase for a number of further dives across the whole data set was also performed to insure the selected thresholds were robust for a number of dive profile shapes (e.g. dives with an active swim phase encompassing a variety of depths, of which there were few in the randomly selected sub-sample). The active swim phase begun at the first point the vertical gradient of the dive fell below 1.05 and ended at the last point at which the vertical gradient rose above -0.2. U-shaped dives were defined as those with an active swim phase of at least four seconds [2] which was also validated against the sub-sampled manually assigned dataset.

To account for potential changes in gradient threshold because of sampling rate, and to keep allocations consistent between tag types, depth data from the CEFAS tags were resampled at a rate of one second to give a dummy dataset representative of the sampling rate of the LOTEK loggers but that could be directly compared to higher resolution data. These resampled profiles were then used to determine appropriate thresholds that gave similar allocations to those obtained using the higher resolution data. The active swim phase begun at the first point the vertical descent gradient of the bird fell below 1.05 and ended at the last point at which the vertical descent gradient was above -0.55. U-shaped dives were defined as those with an active swim phase exceeding 3 seconds which gave more consistent results with the higher resolution data than a 4 second threshold.

| 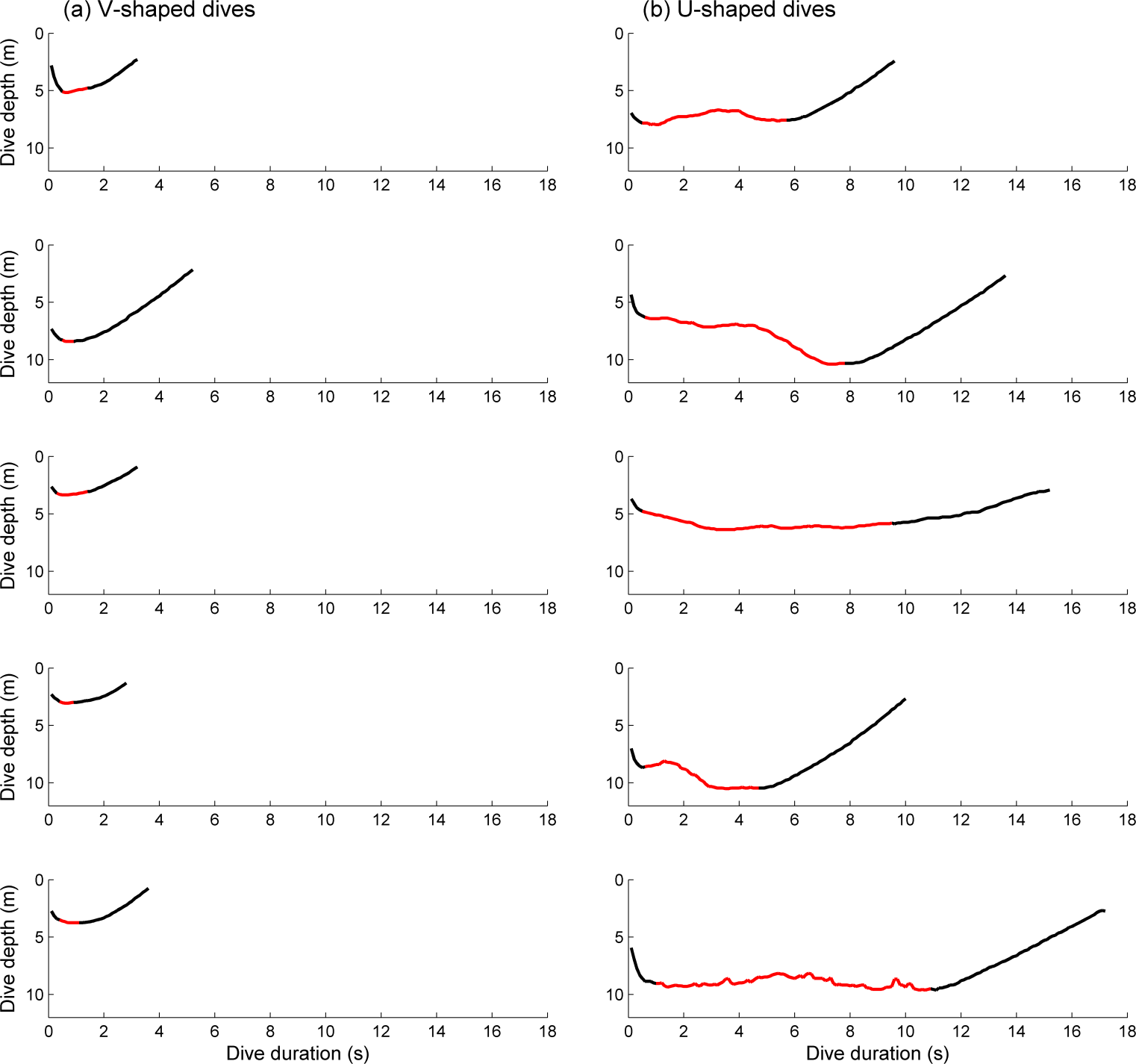 |
| --- |
| Figure S1. Examples of typical dive profiles form the CEFAS tags. Defined active swim phase is highlighted in red. Right hand column (a) plots show dive classified as V-shaped and left hand column (b) plots show those classified as U-shaped. |

**S.2 Habitat use-availability analysis: generation of pseudo-absence locations**

Habitat use was compared to habitat availability to determine if gannets preferentially targeted fronts for diving, and hence foraging [3,4]. The 95% utilisation distribution (UD) of the population sample was calculated using a kernel analysis conducted with the R package adehabitatHR [5] on the GPS locations of all individuals tracked across the two years (Figure S2.a). A binomial response variable (0/1) was then generated. For each dive event (coded as 1), the locations of five pseudo-absences (coded as 0) were randomly selected within the bounds of the 95% UD (Figure S2.b). To allocate front metrics generated using seven day composites to each pseudo absence location, a date was assigned that corresponded to the date of the paired dive event.

| 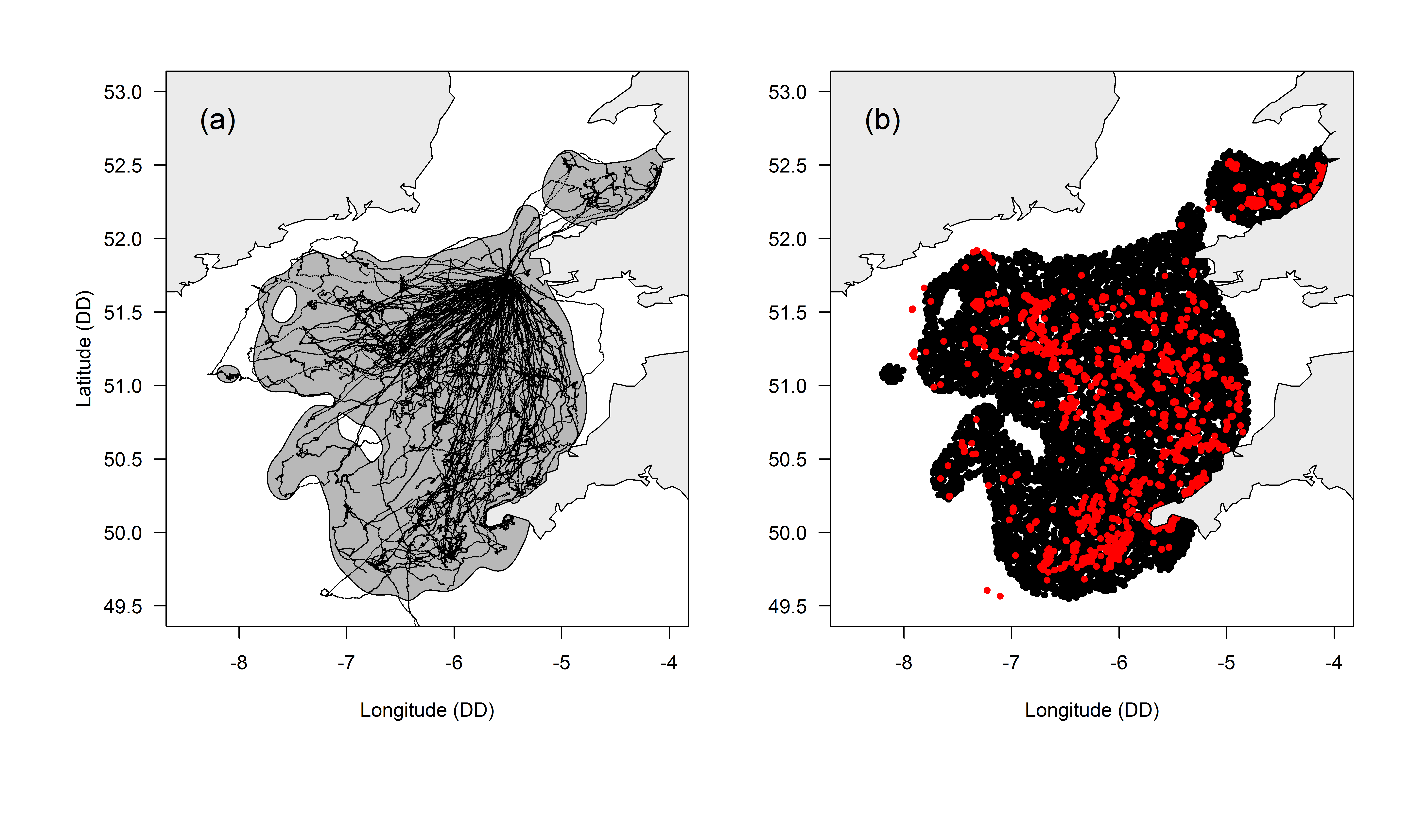 |
| --- |
| Figure S2. From left to right: (a) the 95% utilisation distribution (dark grey shaded area) as calculated using kernel analysis of all foraging trip tracks (indicated by black markers), and (b) the locations of randomly generated pseudo-absences (black markers) and recorded dive events (red markers). |

**S.3 Dive durations**

| 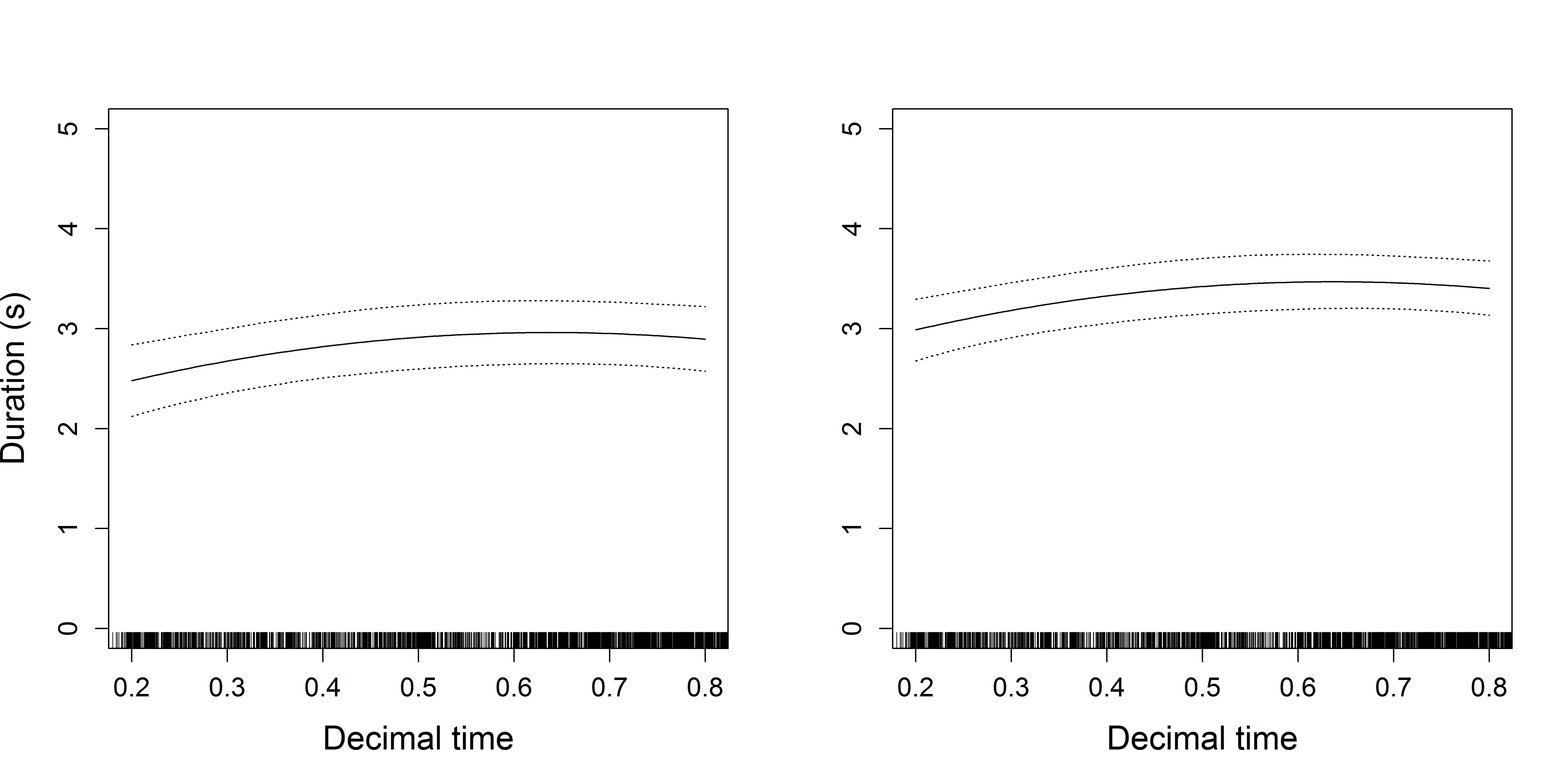 |
| --- |
| Figure S3. Predicted durations of V-shaped dives with time of day (*DecTime*). From right to left; (a) V-shaped dive duration of males and (b) V-shaped dive duration of females. Both show significant increases in dive duration around the middle of the day. Dives were shortest at dusk and dawn. The filled line shows expected dive durations for an ‘average’ bird. Dotted lines show bootstrapped 95% confidence intervals. |

**S.4 References**

1. Ropert-Coudert Y, Daunt F, Kato A, Ryan PG, Lewis S, Kobayashi K, et al. Underwater wingbeats extend depth and duration of plunge dives in northern gannets *Morus bassanus*. J Avian Biol. 2009;40:380–7.

2. Garthe S, Benvenuti S, Montevecchi WA. Pursuit plunging by northern gannets (*Sula bassana*) feeding on capelin (*Mallotus villosus*). Proc R Soc Biol Sci. 2000;267:1717–22.

3. Aarts G, MacKenzie ML, McConnell B, Fedak M, Matthiopoulos J. Estimating space-use and habitat preference from wildlife telemetry data. Ecography. 2008;31:140–60.

4. Cleasby IR, Wakefield ED, Bodey TW, Davies RD, Patrick SC, Newton J, et al. Sexual segregation in a wide-raning marine predator is a consequence of habitat selection. Mar Ecol Prog Ser. 2015;518:1–12.

5. Calenge C. adehabitatHR. 2014. R package v0.4.11
